# Measuring and explaining mortality in Dutch hospitals; The Hospital Standardized Mortality Rate between 2003 and 2005

**DOI:** 10.1186/1472-6963-8-73

**Published:** 2008-04-03

**Authors:** Richard Heijink, Xander Koolman, Daniel Pieter, André van der Veen, Brian Jarman, Gert Westert

**Affiliations:** 1 National Institute for Public Health and the Environment, Bilthoven, The Netherlands; 2 Erasmus University Medical Center, Rotterdam, The Netherlands; 3 Prismant, Utrecht, The Netherlands; 4 De Praktijk Index, Utrecht, The Netherlands; 5 Dr Foster Unit, Imperial College, London, UK; 6 Tilburg University, Tilburg, The Netherlands

## Abstract

**Background:**

Indicators of hospital quality, such as hospital standardized mortality ratios (HSMR), have been used increasingly to assess and improve hospital quality. Our aim has been to describe and explain variation in new HSMRs for the Netherlands.

**Methods:**

HSMRs were estimated using data from the complete population of discharged patients during 2003 to 2005. We used binary logistic regression to indirectly standardize for differences in case-mix. Out of a total of 101 hospitals 89 hospitals remained in our explanatory analysis. In this analysis we explored the association between HSMRs and determinants that can and cannot be influenced by hospitals. For this analysis we used a two-level hierarchical linear regression model to explain variation in yearly HSMRs.

**Results:**

The average HSMR decreased yearly with more than eight percent. The highest HSMR was about twice as high as the lowest HSMR in all years. More than 2/3 of the variation stemmed from between-hospital variation. Year (-), local number of general practitioners (-) and hospital type were significantly associated with the HSMR in all tested models.

**Conclusion:**

HSMR scores vary substantially between hospitals, while rankings appear stable over time. We find no evidence that the HSMR cannot be used as an indicator to monitor and compare hospital quality. Because the standardization method is indirect, the comparisons are most relevant from a societal perspective but less so from an individual perspective. We find evidence of comparatively higher HSMRs in academic hospitals. This may result from (good quality) high-risk procedures, low quality of care or inadequate case-mix correction.

## Background

It is well-known that hospital quality varies widely, yet it remains difficult to measure. In the past, various studies tried to measure health outcomes as measures of hospital quality [1-10]. The most accurately and completely registered outcome seems to be mortality.

A comparison of hospital mortality between hospitals does not show hospital quality directly, because the number of hospital deaths is likely to be influenced by the characteristics of admitted patients. These characteristics will not be distributed evenly across hospitals. Consequently, hospitals that treat more severe patients will have higher expected mortality irrespective of their quality. A thorough analysis of hospital mortality requires case-mix adjustment, for example for differences in diagnosis, age and sex [2]. A popular comparable measure is the hospital standardized mortality ratio (HSMR), which is an indicator that corrects hospital mortality for case-mix differences. It is based on routinely collected medical data.

The main purpose of the HSMR is to give an indication of the quality of care in hospitals. Whether risk-adjusted mortality rates reflect differences in quality of care was studied on various occasions [3]. Since 1999 the HSMR has been used and debated in the UK [4,11-13]. The measure is now used in the US, Canada and Australia to assess care, to identify areas for possible improvement and to monitor performance over time. In the UK some hospitals with a high HSMR initiated organizational changes and were able to improve their risk-adjusted mortality scores [6,7]. Furthermore, some studies found a relationship between quality indicators and hospital standardized mortality [8-10] indicating that HSMR figures can be used as indicators of hospital quality.

It would be useful for hospitals and health policy makers to investigate variables that are associated with HSMR variation. This will enhance the insight into the variation in hospital outcomes and may lead to more specific research questions. Hospitals, for example, behave differently with respect to patient transfers or discharge procedures, which may influence their performance with respect to the HSMR. Other contextual variables that might influence the HSMR should be examined too, for example hospital doctors per bed or General Practitioners (GPs) per head of the population [4].

Health outcomes have been used in quality-of-care research, because they have intrinsic value. In addition, an increasing number of indicators (such as mortality scores) has been made public, especially in the UK and the USA [1,14]. These public indicators can influence outcomes of health care by informing consumer choices and consumer behavior, by motivating quality improvements through affected reputation, and by inherently setting professional standards [15]. As a result of this, hospitals are increasingly held accountable for their performance [1,14].

Against this backdrop, it is important to have useful and accurate performance measures [14]. Performance variables should at least be corrected for differences in case-mix as with the HSMR. Otherwise hospitals may be penalized for bad outcomes that are actually outside their control. As hospitals are increasingly judged on these types of measures it will be very useful, for policy makers and hospitals, to gain further insight into the HSMR.

The goal of this paper was to explain the variation in HSMR scores within and between hospitals using factors that can and cannot be influenced by the hospital. Therefore, we first explain the estimation of the HSMR and its interpretation in the Data section. Then we clarify our explanatory multilevel model that uses the yearly HSMRs at the lowest level.

## Methods

### Data

The Dutch HSMRs were calculated using hospital episode statistics from 2003 to 2005 that are recorded in the National Medical Registration (Landelijke Medische Registratie). Within this system all hospital admissions (day cases and in-patient cases) are registered, including variables such as age, gender, diagnosis and length of stay. Seven out of 101 hospitals were excluded in all years because of insufficient registration. For 2005, another two hospitals were missing because of unavailable mortality data.

All environmental characteristics were calculated for 'WZV-regions' in which hospitals reside. The country is divided by law (WZV-law) in 27 health regions. Data from GP-registries, collected by the Netherlands institute for health services research (Nivel), were used to calculate *local number of GPs per 10,000 inhabitants*. Average *Social Economic Status *(SES) scores in each region were computed by the Social and Cultural Planning Office (SCP). Finally, the *local number of nursing home beds per 10,000 inhabitants *was obtained from registries kept by Prismant.

Hospital characteristics data were available from an obligatory, yearly hospital survey conducted by Prismant. This survey involved all Dutch hospitals; three hospitals failed to provide any hospital data. These were excluded in the explanatory HSMR analysis, which finally included 89 hospitals. All hospital and environmental characteristics, except *discharge procedure *and *year*, were available for one year only. Therefore, it was assumed that variables available for one year were constant between 2003 and 2005. This assumption seems realistic, because the Dutch hospital sector has been rationed for many years and the government has controlled hospital size, volume and teaching status.

### HSMR

The dependent variable in this study was the HSMR. It was calculated on a year by year basis for all Dutch hospitals. The HSMR compares the actual number of hospital deaths to the expected number. To select patients we used their primary diagnosis within the diagnostic groups (coded using Clinical Classification System, CCS) that nationally account for 80% of all in-hospital deaths. Both day cases and in-patient admissions were included in the analysis.

While the HSMR was originally based on indirect standardization, at present binary logistic regression is used to estimate expected deaths based on the national population. Logistic regression allows the use of continuous variables and gives researchers the freedom to disregard interactions when none are believed to exist. This helps to build a parsimonious model. For the estimation of the HSMR this characteristic is believed to compensate for the disadvantages of parameterization. In practice both approaches provide similar results as they are asymptotically equivalent. The HSMR is equal to the ratio of actual deaths to expected/predicted deaths (×100). This can be interpreted as an adjusted hospital mortality ratio which takes case-mix into account. On a national level hospital mortality was statistically significantly associated with: primary diagnosis, age, sex, admission urgency (urgent/not-urgent or emergency/elective (planned)) and length of stay (LOS), for each of the diagnoses leading to 80% of all deaths. The primary diagnosis is the main diagnosis that led to the admission, but not necessarily the diagnosis that caused death. These national risk-of-death rates, stratified by diagnosis, age, sex, urgency and LOS were applied upon each hospitals population to calculate expected deaths. The national HSMR for the benchmark year is 100 by definition. Because national risk-of-death rates are applied upon each hospitals population, an HSMR significantly higher than 100 indicates that the hospital's death rate is higher than if *its *patients had national mortality rates. We used the HSMRs of 2003 to benchmark later years.

By comparing expected deaths with actual deaths using a regression model we mimic indirect standardization. Both techniques use the hospital population itself as the reference population, as this is the population to which the category specific reference rates were applied. Therefore, a different case-mix distribution was used for each HSMR. This provides the best mortality score from a societal perspective as it is based on the population the hospital actually serves, not the national reference population. This stimulates each hospital to do well for each patient equally, and not to focus on those patients that are rare compared to the national population and consequently receive a high weight (which would be the case if the HSMR was based on direct standardization). From an individual perspective, the HSMR may not provide the information patients are after, because *irrespective of his or her characteristics *he or she may be better off in a hospital with a higher HSMR. Information for patients should therefore be based on *direct *standardization.

### Environmental characteristics

*The local number of GPs per 10,000 inhabitants *was included, because it was found to be negatively associated with the HSMR in other studies [4]. In regions with a lower number of GPs, GPs may experience a higher workload and have a less effective risk-management of their patients. It was also suggested that this high workload could result in the delivery of more emergency admissions to hospitals [4]. The HSMR calculation was however corrected for the urgency of the admission. On the other hand, GPs with a high workload might refer patients to a hospital sooner and deliver a healthier population to the hospital. This would suggest a positive relation between the number of GPs in the region and the HSMR.

Hospitals in regions with a relatively high/low proportion of people in low *Social Economic Status *(SES) groups may get higher/lower HSMR scores [16,17]. Regionally defined socio-economic conditions are outside the control of the individual hospital. Per region an average SES score (between -1 and 1) was calculated, based on income, unemployment rates and education.

*The local number of nursing home beds per 10,000 inhabitants *is another indicator that could influence the HSMR [5]. If there is a shortage of nursing homes in a certain region, hospitals may, unnecessarily, need to take care of patients that should be in nursing homes. This could generate higher or lower HSMRs.

### Hospital characteristics: organizational form

First a distinction was made between two *hospital types*: academic and non-academic hospitals. The HSMR might not be able to pick up all variation in patient severity related to hospital type. Dutch academic hospitals presumably get more severe cases. Furthermore, non-academic hospitals may transfer the most severe cases to academic hospitals. These effects may result in higher HSMRs for academic hospitals.

*Teaching status *is another hospital typification often used in studies about hospital performance [9,16-20]. Presumably, teaching hospitals have higher quality personnel resulting in better outcomes. On the other hand personnel in teaching hospitals may experience more pressure, because of extra teaching activities, resulting in worse health outcomes. Results, however, have not been consistent over the years and vary among conditions [21].

Finally number of beds was used as a proxy for *hospital size*.

### Hospital characteristics: process measures

It is often assumed that *volume *is inversely related to mortality [22]. High-volume hospitals, performing treatments more often, are able to generate lower mortality rates compared to lower volume hospitals [22-24]. In this study the *number of patients per bed *was used as proxy for volume.

*Discharge procedure *was included, because hospitals may influence mortality rates through their discharge procedures. If a hospital discharges a relatively large proportion of its patients (alive) to other health care institutions and lets them die in these other institutions, it can reduce its HSMR without having higher quality health care. A dummy variable was set up to account for this. First, the percentage of all discharges to other institutions was calculated. Second, hospitals with above average rates received a value of one and hospitals with below average rates received a value of zero.

The *bed occupancy rate *could influence the HSMR score too. Occupancy rates were found to be positively related to hospital mortality [25,26]. A high occupancy rate may create more pressure upon the hospital personnel resulting in overwork. Having less time for each patient may influence treatment outcomes negatively. The bed occupancy rate was calculated as: actual number of bed days/(available beds*365).

### Hospital characteristics: inputs

Finally we included some of the inputs (in terms of labour) used by hospitals. The amount of personnel per bed possibly influences hospital mortality [4,18]. Numbers of *doctors per bed *and *nurses per bed *were included in the analysis. It has been found that the number of doctors per bed is inversely related to hospital mortality [4]. The number of *nurses per bed *may influence quality and hospital mortality too. Having more personnel per bed could increase the quality of care and lower the HSMR. Both 'input-variables' may experience diminishing returns: at a certain point the marginal benefit (lower mortality) of an extra nurse decreases.

## Analysis

### Time trend

The first goal of this study was to assess the variation in HSMR scores within hospitals over time and between hospitals. A two-level multilevel model was used to make use of the hierarchical structure of the data. We assumed that the longitudinal observations were correlated within each hospital. In this way a two-level model was created: hospital data for each year at level one (year denoted by t) and average hospital data at level two (hospital denoted by i):

*y*_*ti *_= *α *+ *βx*_*ti *_+ *υ*_0*i *_+ *υ*_1*i*_*x*_*ti *_+ *ε*_*ti*_

where *y*_*ti *_reflects the estimated HSMR for hospital i at time t. The part '*α *+ *βx*_*ti*_' equals the fixed part of the model consisting of the mean of the intercept *α *and the regression coefficient *β *that is constant for all years and is multiplied by the variable year, *x*_*ti*_. The random part of the model, '*υ*_0*i *_+ *υ*_1*i*_*x*_*ti *_+ *ε*_*ti*_', reflects level-two residuals *υ*_0*i *_and *υ*_1*i *_and level-one residual *ε*_*ti*_. Level-two residuals represent variation between hospitals and level-one residuals represent variation between years. The residual *υ*_0*i *_is the random intercept, arising from a normal distribution and describing the deviation of hospital i from the average intercept. We added a random slope, *υ*_1*i*_, to allow for random variation in the relationship between HSMR and year across hospitals. The variable year was centered in order to test the relationship between random intercepts and random slopes [27]. The variance of the random slope and the covariance of the random slope and intercept were tested and found to be significantly different from zero. The residual *ε*_*ti *_describes the unexplained variation at the lowest level (year). We assumed a constant association between time and outcome. More flexible specifications did not improve model fit significantly. The correlation of observations per hospital was tested with the Intraclass Correlation Coefficient (ICC). The ICC is defined as the ratio of the between hospital variance and the total hospital variance, formally $\frac{\sigma_{u0}^{2}}{\sigma_{u0}^{2}+\sigma_{e}^{2}}$[28].

### Explanatory analysis

Initially bivariate Pearson correlation coefficients and univariable regressions were calculated between the HSMR and the above mentioned variables. In addition, multivariable regression models were used to model the hypothesized relations. First, the multivariable regression was performed using pooled Ordinary Least Squares (OLS) regression, including a correction for clustering. Second, two-level Hierarchical Linear Models were used; one model including all variables, and the other including only variables that were significantly correlated with the HSMR in univariable regressions. The multilevel method allowed us to assume that the longitudinal observations were clustered within each hospital (as in the time-trend model). Similar to the time trend model two levels were created with hospital at level two and year at level one, which yielded

*y*_*ti *_= *α *+ *β *_0_*X*_*ti *_+ *β *_1_*Z*_*i *_+ *υ*_*i *_+ *ε*_*ti*_

where *y*_*ti *_enotes the estimated HSMR for hospital i at time t. The fixed part of the model '*α *+ *β*_0_*X*_*ti *_+ *β*_1_*Z*_*i*_', consists of the mean of the intercept *α*, the coefficients *β*_0 _for a vector of variables at level one *X*_*ti *_(*year *and *discharge procedure*), and the mean of the coefficients *β*_1 _for a vector of variables *Z*_*i *_at level two (all other explanatory variables). The random part of the model, '*υ*_*i *_+ *ε*_*ti*_' reflects level-two residual *υ*_*i *_and level-one residual *ε*_*ti*_. The residual *υ*_*i *_is the random intercept, arising from a normal distribution and describing the deviation of hospital i from the average intercept. Random slopes were tested for all explanatory variables but none of the variances was significantly different from zero. The residual *ε*_*ti *_describes the unexplained variation at the lowest level. Cross-level interactions were also tested (e.g. between *hospital type *and *year*) to consider different trends in HSMR for different independent variables. At 0.05 level, none of the interaction terms was significantly different from zero. All models were estimated using MLwiN software (version 2.02).

## Results

### Descriptives

We present descriptive statistics in Table 1. The total number of in-hospital deaths decreased between 2003 and 2005. The variation in HSMR measured in standard deviations varied between 16.2 and 14.3. In all years the hospital with the highest HSMR had an HSMR score about 1.5 times as high as the average score and about twice as high as the lowest score. As these could be sensitive to outliers we also divided the average HSMR of the worst five hospitals by the average HSMR of the best five hospitals. This resulted in a ratio of 1.85.

**Table 1:** Descriptive statistics of mortality in Dutch hospitals between 2003 and 2005

	2003	2004	2005
Total deaths	34,391	32,408	31,808
			
*(a)*			
HSMR Mean (SD) and all hospitals	100 (14.9)	90 (14.5)	83 (11.9)
HSMR Mean (SD) 7 academic hospitals	117 (20.1)	103 (23.3)	94 (16.9)
Min/Max HSMR	74 – 151	62 – 140	57 – 120
			
*(b)*			
HSMR Mean (SD) all hospitals	100 (14.9)	100 (16.2)	100 (14.3)
Min/Max HSMR	74 – 151	69 – 156	70 – 144

(a) HSMR between 2003 and 2005 (average 2003 = 100).

(b) HSMR between 2003 and 2005 with average HSMR set at 100 each year.

Furthermore, we looked at the relative position of each hospital over time. The position of hospitals can change over time and a significant switch in positions could indicate big changes in relative quality of hospitals. Alternatively, this finding could indicate poor reliability of the HSMR. The Spearman's rank-correlation, correlating the HSMR scores for 2003–2005, showed a significant positive relationship of 0.74 between 2003 and 2004 and of 0.76 between 2004 and 2005. Most hospitals with a high (low) HSMR in 2003 (2004) also had a high (low) HSMR in 2004 (2005). It demonstrates that besides a rather stable dispersion, individual hospitals also had stable relative positions in these years.

### Time trend

Model 1 was used to examine the trend in HSMR scores. Table 2 demonstrates the results of the time-trend (multilevel) model and shows that the HSMR followed a constant decreasing trend over time. It also shows that most of the variation in the HSMR was caused by variation between hospitals rather than variation within hospitals over time (reflected by the ICC). This finding is often used to justify the use of a multilevel model, assuming correlated observations, per hospital, over time. The negative covariance shows that hospitals with a higher intercept had a greater decrease in HSMRs.

**Table 2:** Results Model 1^1^

Constant	99.0 (1.4)
Year	-8.4 (0.5)*
	
n	280
	
*Level 1 variance*	42.8 (6.3)
*Level 2 variance*	
Random intercept for hospitals	184.0 (32.5)
Random slope for hospitals	9.6 (5.5)
Covariance random intercept and random slope	-26.5 (10.4)
	
ICC	0.81
-2*loglikelihood (IGLS)	2098

^1 ^Coefficients are shown with standard errors between brackets.

* Statistically significant (95% interval).

### Explanatory analysis

The association between HSMRs and environmental and hospital characteristics was studied next. The results are presented in Table 3. The univariable correlations show that, besides the time variable, *GPs per 10,000 inhabitants*, *hospital type*, *hospital size*, *volume *and *percentage of hospital days for day cases *were significantly correlated with the HSMR. The correlations of these variables also had the expected signs. Columns five to seven show the results of the multivariable regressions. Column five and six show the results of the multilevel analysis. The seventh column shows the results of the pooled OLS with a correction for clustering. The results were fairly similar in both models.

**Table 3:** Results Model 2^1^

	Mean (SD)	Corr.	Regression coefficient (standard error)			
			Univariable	Multilevel	Multilevel All^2^	Pooled OLS All^2^

*Level 1*						
Year	-	-0.45*	-8.4 (0.5)*	-8.2 (0.5)*	-8.3 (0.5)*	-8.3 (0.6)*
Discharge procedure	-	0.04	0.7 (2.3)	-	1.0 (1.9)	-0.7 (2.5)
						
*Level 2*						
GPs per 10,000 inhabitants	5.3 (0.3)	-0.17*	-8.1 (3.9)*	-10.6 (3.8)*	-10.5 (3.9)*	-10.2 (3.9)*
SES	0.3 (0.4)	-0.04	-1.7 (3.5)	-	-2.1 (3.4)	-1.9 (3.2)
Nursing home beds per 10,000 inhabitants	39.0 (7.1)	-0.02	-0.0 (0.2)	-	-0.0 (0.2)	0.0 (0.2)
						
Hospital type	-	0.26*	15.1 (4.7)*	14.7 (5.9)*	14.5 (6.1)*	15.5 (7.6)*
Teaching status	-	-0.02	-1.1 (2.7)	-	-4.8 (3.1)	-5.3 (3.0)
Hospital size	483 (245)	0.18*	0.01 (0.0)*	-0.0 (0.0)	0.0 (0.0)	0.0 (0.0)
						
Volume	36.8 (5.3)	-0.21*	-0.6 (0.2)*	-0.2 (0.3)	-0.1 (0.3)	-0.1 (0.2)
Bed occupancy rate (%)	65.0 (8.8)	0.04	0.1 (0.2)	-	-	-
Beddays for daycases/total beddays (%)	12.6 (2.8)	-0.25*	-1.4 (0.5)*	-0.6 (0.5)	-0.6 (0.5)	-0.7 (0.5)
Nurses per bed	1.1 (0.2)	0.10	7.1 (6.3)	-	-	-
Doctors per bed	0.3 (0.1)	0.05	6.8 (12.3)	-	-	-
						
N	-	-	-	271	267	267
ICC	-	-	-	0.66	0.66	-
-2*loglikelihood (IGLS)	-	-	-	2021	1990	-

^1 ^Y = HSMR (2003 = 100), Corr. = Bivariate Pearson correlation coefficient.

^2 ^Due to perceived multicollinearity occupancy rate, doctors/bed and nurses/bed were excluded.

* Statistically significant (95% interval)

The model in the fifth column included all variables that were significantly correlated with the HSMR (see column three and four). It indicates that the coefficients of the variables *year*, *GPs per 10,000 inhabitants *and *hospital type *were all significant. When corrected for the former variables, the variables *hospital size*, *patients per bed *and *percentage of days in day cases *were no longer significantly related to the HSMR. The sixth column shows the multivariable regression including all variables, besides the ones excluded due to perceived multicollinearity. Excluded were *doctors per bed*, *nurses per bed *and *bed occupancy rate *(which correlates strongly with *patients per bed*). Like the results in the fifth column only *year*, *GPs per 10,000 inhabitants *and *hospital type *remained significantly related to the HSMR. There does not seem to be any association between the hospital inputs *doctors per bed *or *nurses per bed *and the HSMR scores. The same is true for other variables, such as *discharge procedure*.

## Discussion and Conclusion

On average, HSMR scores in the Netherlands declined between 2003 and 2005. The variation between hospitals, however, remained substantial (approximately 1.8 higher HSMR scores for the worst-five compared to the best-five hospitals). Furthermore, most hospitals maintained a stable relative position between 2003 and 2005, which suggests that the reliability of the HSMR is good. The explanatory analysis showed that the variables *year*, *GPs per 10,000 inhabitants in the hospital region *and *hospital type *were significantly associated with the HSMR.

In the literature various predictors of hospital mortality have been studied [3,4,8-10,13,16-26,29,30]. The goal of this paper was to explain (between and within) variation in new Dutch HSMRs for the first time. In doing so, we were able to place Dutch results in an international perspective. Furthermore, we used multilevel modeling to account for the hierarchical structure of the data. Finally, we clearly explained the possibilities of HSMR scores: they can be useful from a societal perspective and they should not be used from a patient perspective.

The results should be interpreted with a number of study limitations in mind. First, the dataset used to calculate HSMR scores was based upon hospital episodes (an admission followed by a discharge) and not upon patients. Several episodes may involve one patient. Hospitals may have different policies regarding the number of episodes per patient, which influences the number of registered episodes. This could affect the HSMR score without reflecting differences in quality. Second, case-mix correction through the Dutch HSMR model may not capture all case-mix differences. Mortality was corrected for age, sex, primary diagnosis, length of stay and admission urgency. However, especially for secondary diagnoses, it was unknown whether specific comorbidities were present. Still, Aylin et al. [31] argue that routinely collected administrative data (such as our data) can produce valid case-mix corrected measures of hospital mortality. A final consideration could be made with respect to the inputs. Remarkably, the labour input data did not explain any HSMR variation. It may well be possible that a further distinction between different types of labour or different personnel qualifications will give us more information and may in fact explain some of the variation.

The results and considerations show that the HSMR needs to be studied carefully, before making it public or incorporating it in policy decision making. Variation between hospitals would indeed seem to point at systematic differences in processes between hospitals leading to systematic HSMR variation. This is underlined by the ICC, which showed relatively large between-hospital variation.

What is notable here is the – on average – high HSMR for academic hospitals. Various explanations are possible. First, academic hospitals may perform more high-risk procedures which have a higher risk of death. These high-risk procedures may combine better health outcomes with higher risk of acute death. Therefore, they could be considered high quality care that causes higher HSMRs. Consequently, high HSMRs can result from good quality of care. Second, with respect to mortality, academic hospitals may perform worse than the others. This could happen as a result of organizational deficiencies. Academic hospitals may be too large, inefficient or have more inexperienced doctors. Table 3, however, shows that size hardly influenced the HSMR, and having inexperienced doctors (teaching status) did not have the sign to support this conclusion. Third, we may not have captured all the case-mix differences; rendering an HSMR comparison with other hospitals invalid. Model misspecification could be due to measurement errors, misspecified functional forms and omitted variable bias. One example of such an omitted variable is the readmission rate per hospital. Hospitals with high readmission rates may have more severe patients. However, the variable *readmissions *was not included due to underreporting.

While the third cause calls for an improved standardization of the HSMR, the other two causes do not. Good quality high-risk care will lead to better outcomes on other indicators of quality of care, and they remind us that no indicator will fully capture quality of care. For that goal we need global measures, not indicators. Moreover, the choice to provide high-risk care can be influenced by the hospital and therefore is no environmental factor. This also holds for organizational deficiencies. Further research should indicate which of the three explanations mentioned above contributes to the variation in HSMRs we observe and to what extent. Such research is required as without it we cannot rule out the possibility of incomplete standardization that is required to compare all hospitals.

Another remarkable result is the influence of the number of GPs in the hospital region. The presence of more GPs in the region is associated with a lower HSMR. This relationship was also found in the UK [4]. This may confirm the hypothesis that in areas with relatively few GPs, GPs may experience a heavy workload. This could result in worse risk-management performance, affecting the health of the patients sent to the hospital. Alternatively, GPs may be less prone to settle in less attractive areas, and whatever makes these areas less attractive could lead to higher HSMRs.

In addition to global outcome measures, outcome indicators such as the HSMR clearly are indicators of interest. We argue that the HSMR can be a useful indicator to monitor hospital performance over time and to compare hospital performance between hospitals. While the HSMR is suited for that goal, it is estimated using varying populations and thus is not directly usable for individual prospective patients to choose a hospital.

## Competing interests

The author(s) declare that they have no competing interests.

## Authors' contributions

RH designed the study, performed the analyses and wrote the draft manuscript. XK and GW participated in discussing the design and analysis and in revising the manuscript. DP acquired and provided the data. DP, ADVD and BJ assisted in discussing the results and revising the manuscript. All authors read and approved the final manuscript.

## Pre-publication history

The pre-publication history for this paper can be accessed here:


